# Green Synthesis of Nanoparticles Using Bio-Inspired Systems and Electrically Conductive Pattern Fabrication through Laser-Direct Writing

**DOI:** 10.3390/nano12030545

**Published:** 2022-02-05

**Authors:** Sangmo Koo

**Affiliations:** Department of Mechanical Engineering, Incheon National University, Incheon 22012, Korea; skoo@inu.ac.kr

**Keywords:** tryptophan, laser-direct writing, photoreduction, nanoparticle, sintering, flexible, bio-inspired system

## Abstract

Systems existing in nature have evolved to operate efficiently over a long period of time, enabling efficient material transformation and processing. These natural systems provide hints for the synthesis of metal nanoparticles through efficient electron generation and transport towards metal ions for the reduction process. In this study, based on the efficient electron transfer mechanism between tryptophan (Trp) in the living body, the possibility of advanced silver patterning on flexible substrates has been presented through laser-direct writing. Irradiation of a low-power laser on the precursor induces the reduction of silver ions to nanoparticles. The sintering of these generated nanoparticles induces a silver conductive pattern by a photothermal/chemical reaction. The method of this study has strength as it supports the possibility of conductive pattern fabrication on various substrates (e.g., glass and PDMS) using a silver-based organic ink with low laser power compared to the conventional nanoparticle-based sintering method. It also suggests its suitability to various applications in terms of sophisticated pattern fabrication with minimized substrate denaturation.

## 1. Introduction

Due to the rapid growth in nanotechnologies and extensive applications in various fields, significant concerns about environmental impacts, such as the recycling of nano products, have emerged regarding the toxicity during synthesis and post-processing [1,2]. For the formation of nanoparticles, metal nanoparticles can be synthesized using photoreduction [3,4], laser ablation [5], chemical reduction in aqueous media [6] (which is composed of other polymer surfactants and reducing agents), and compound reduction in soft/solid matrices. However, toxic substances are required, or hazardous materials can be generated, during the process [7]. From this point of view, the green synthesis of metal nanoparticles using nature-derived materials is attractive to the next generation of synthesis methods due to the use of soluble, non-toxic chemicals, which are environmentally friendly, cost-effective, and relatively reproducible. For example, the synthesis of nanomaterials has been conducted using biological plants and microorganisms (e.g., plants, algae, fungus, yeast, bacteria, and viruses) [8,9,10,11,12,13]. In particular, numerous chemical/physical synthesis methods for silver nanoparticles that use a non-toxic method in an aquatic environment were proposed [10,14]. In addition, studies on the morphology control of synthesized nanoparticles have been performed [15].

The synthesis of nanoparticles should be considered using an eco-friendly synthesis method, and three main factors should be considered: (i) selection of solvent, (ii) selection of reducing agents, and (iii) selection of a non-toxic stabilizing agent to stabilize the silver nanoparticles [16]. The composition of the precursor material used in this study was based on naturally derived materials, except for the silver ion. 

First, water was used as a solvent in this study. Water is biocompatible, and a representative non-toxic material among various organic solvents [17]. The reducing agent, tryptophan (Trp) was used. Trp is an amino acid that is necessary for the synthesis of vitamin B3 (niacin), a precursor of serotonin, and a neurotransmitter. Due to the chemical structure of Trp, it was oxidized and generated one electron corresponding to an oxidized radical as laser irradiation. This de-protonation rapidly induced the formation of the tryptophan indolyl radicals in the solution [18]. Moreover, Trp has an aromatic structure, which caused P-P stacking, and accelerated electronic transfer due to proton-coupled electron transfer.

Polymers have been used as a matrices and stabilizers of nanoparticles. Among these polymers, gelatin was introduced as a non-toxic stabilizer for generated nanoparticles. The stabilization of nanoparticles is an important factor for successful patterning. Gelatin serves to minimize the oxidation, aggregation, and precipitation of the nanoparticles through nanoparticle-composite formation, thereby making it possible to maintain the stability of the nanoparticles [19,20]. Gelatin has a random coil form and exhibits a reversible gel-solution state conversion below normal body temperature. This reversible property allows it to work as a stabilizer [21]. As the temperature rose, the gelatin polypeptide chain was mainly in the form of a flexible, unfolded coil in a solution. However, it could be partially reconfigured as an ordered α-helix in the form of a 3D polypeptide when cooled to room temperature through a self-assembly process. During this process, the microdomain could be generated in the gelatin, and this microdomain stabilized nanoparticles by coexistence with a nanoparticle [22]. The gelatin polypeptide chain also performed a role as a template in the formation and growth of the nanoparticles, which allowed the nanoparticle sizes to be controlled. It also contributed to the nanoparticles being dispersed uniformly. Moreover, in terms of synthesis, the presence of the –NH_2_, –SH, –COOH functional groups of gelatin and suspended double bonds may have induced a reduction of metal ions into nanoparticles. Therefore, gelatin plays various roles in the stabilization, dispersion, and synthesis of nanoparticles [21]. As gelatin is homogeneous and optically transparent, its concentration should be optimized.

Hence, combining this precursor with a laser-based fabrication technique solidified the meaning of green engineering. The laser-based machining not only produced high-performance patterning, but it was also a simple, scalable, fast, cost-effective, and precise fabrication method without the use of expensive vacuum equipment and photomasks at room temperature. Moreover, it could shorten the entire processing time due to the simplicity in the design change. Laser-based sintering methods are attracting attention as low-temperature flexible electrode fabrication due to the controllability of thermal transfer. It is possible to directly heat a material by applying laser-thermal energy to a localized region. When nanoparticles are used in the processing process, the melting temperature of the nanoparticles can be reduced to be in the range of 100 to 200 °C due to the thermodynamic size effect. Therefore, metal nanoparticles can be melted in a localized region by laser irradiation, and the metal pattern can be fabricated through the sintering process by surface plasmon resonance with controllable thermal penetration [23,24,25]. As a result, this method reduces the thermal stress applied to the polymer substrate, which is vulnerable to heat. 

In this study, we introduced laser-direct metal patterning using biocompatible materials. It was possible to synthesize silver nanoparticles and pattern the electrode in an eco-friendly way, in that no toxic organic solvent was used and no contaminants were generated. The Trp induced efficient nanoparticle generation through efficient, proton-coupled electron transfer by laser irradiation. The uniform precursor coating on the substrate could be achieved through the increase in appropriate viscosity by adding gelatin into the precursor. Moreover, stabilization of the nanoparticles was achieved using gelatin simultaneously. In this study, we prepared the precursor with an optimal concentration ratio of Trp and gelatin and conducted laser direct writing for a high-resolution, customized electro-conductive metal conductive pattern. Additionally, excellent electrical properties and durability against cyclic mechanical deformation were confirmed.

## 2. Materials and Methods

### 2.1. Preparation of Precursor Material and Characterization

A precursor was prepared to synthesize the silver nanoparticles using a photoreduction process. The precursor was composed of silver nitrate (AgNO_3_, Sigma Aldrich, St. Louis, MO, USA), gelatin (gel strength 300, type-A, Sigma Aldrich, St. Louis, MO, USA), and tryptophan (Sigma Aldrich, St. Louis, MO, USA). Each material was dissolved in deionized (DI) water to prepare aqueous solutions. The tryptophan, which acted as a reducing agent, was prepared at 0.25 g Trp in 10 mL DI water and mixed vigorously for 1 h using a magnetic stirrer. The solution was then filtered using a 0.2-micron filter. The prepared tryptophan aqueous solution was stored in a brown bottle to minimize additional reactions with light. The homogeneous gelatin solution acting as a stabilizer was prepared by dissolving about 2 g gelatin in 8 mL of 60 °C DI water and was mixed by magnetic stirrer for 1 h. To prepare a uniformly dispersed precursor, tryptophan and 1M silver nitrate solutions were mixed with gelatin solution vigorously for 1 h. The appropriate mixing ratio will be discussed in detail in the Section 3. Precursor materials were also stored in a brown bottle to prevent any reactions with external light and stored for 30 min in a vacuum chamber to eliminate the captured gas.

The absorption spectra of the precursor were measured using a UV–vis spectrophotometer (J-5100B, Beijing, China). The nanoparticle sizes were measured with a field emission scanning transmission electron microscope (TEM, Talos F200X, Thermo Fisher Scientific, Waltham, MA, USA) operated at a 200 kV, which provided 0.25 nm point resolution. The distribution of nanoparticle sizes was analyzed using ImageJ (NIH, Bethesda, MD, USA) and MATLAB (Mathworks, Natick, MA, USA) for image processing. High-resolution X-ray diffraction (HR-XRD, SmartLab, Rigaku, Tokyo, Japan) data of the nanoparticles were collected using a diffractometer with a Ni filtered CuKα radiation source (λ = 1.5412 Å) of 45 kV and 200 mA. 

### 2.2. Laser Setup and Pattern Fabrication

The precursor material was coated using a spin coater to obtain a film of uniform thickness. The spin coating speed and time were adjusted to obtain a suitable coating thickness. Before spin coating, the diced glass surface was cleaned using acetone, isopropyl alcohol, and DI water several times. Then, nitrogen (N_2_) gas was blown onto the surface to dry it. 

**Figure 1:**
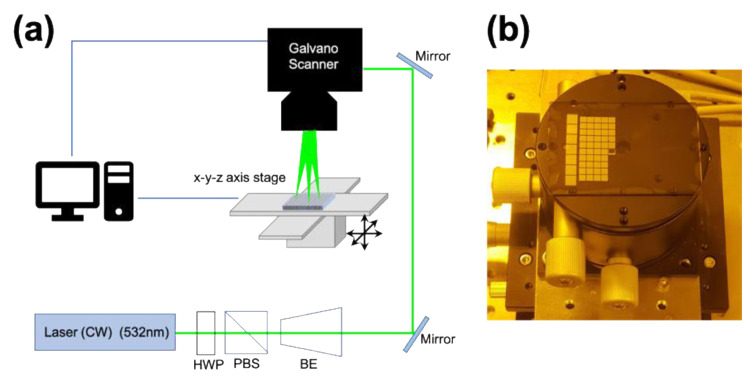
Laser system for sintering. (**a**) Schematic of the experimental setup, and (**b**) image during patterning on the laser system.

The polydimethylsiloxane (PDMS, Sylgard 184, DuPont, Wilmington, DE, USA) film was prepared by mixing the precursor material and the curing agent with a weight ratio of 10:1. Moreover, spin coating was performed for 30 s at 1000 rpm on a glass substrate to fabricate a thin PDMS film. Spin-coated PDMS film was cured in a vacuum oven at 60 °C for 3 h. Moreover, it was cleaned with 70% ethanol and DI water several times and was dried in a vacuum oven at 60 °C after cleaning. The oxygen plasma was applied to improve wettability before precursor material coating. The spin-coated precursor film was mounted on an X-Y-Z stage and aligned perpendicularly to laser irradiation (Figure 1). A pattern was fabricated along a beam path generated by a continuous laser (Finesse pure, Laser quantum) with a central wavelength of 532 nm, with 3W of maximum power. The laser power was adjusted with an attenuator comprised of a half-wave plate (WPH10M-532, Thorlabs, Newton, NJ, USA) and a polarizing beam splitter (PBS, Thorlabs, Newton, NJ, USA). The choice of laser wavelength in laser-based sintering is an important issue, as wavelength is closely related to penetration depth [26]. According to the UV–Vis absorbance data, the precursor could absorb optical energy within a wide range of wavelengths, including 532 nm. In addition, since the thickness of the coating was thin, the thermal energy produced by the laser could be delivered to the substrate in the processing conditions in the 532 nm wavelength. It was also observed that the patterns adhered to the substrate well.

For fabrication of arbitrary shape patterns, a galvanometer scanner (HurrySCAN II, Scanlab, Munchen, Germany) with an f-theta lens (S4LFT5165/292, SILL Optics, Wendelstein, Germany) was adopted and its spot size was approximately 750 μm. The mirror position of the galvanometer scanner was adjusted using software (SAMLight, SCAPS, Oberhaching, Germany). The power of the laser beam was measured using a power meter (S121C, Thorlabs, Newton, NJ, USA) at the inlet of the galvanometer scanner. During the laser writing process, patterning was performed in a dark surrounding environment to prevent any unwanted reactions due to external light. For post-processing, the sample was immersed in DI water for 1 min to remove the un-sintered region, and then air-dried by blowing N_2_ gas.

### 2.3. Pattern Characterization

A surface profiler and a scanning electron microscope (SEM) were used to characterize the shape and morphology of the sintered pattern. To obtain the topography and cross-section profiles of a pattern, the surface profiler (DEKTAK XT-E, Bruker, Billerica, MA, USA) was used. The shape of the pattern and the high-resolution composition mapping was carried out using SEM energy-dispersive X-ray spectroscopy (SEM-EDX, JEOL JSM-780F, Tokyo, Japan). The resistance measurement was performed to verify the connectivity of the sintered metal pattern using a two-probe technique with semiconductor parameter analyzer (HP4155A, Hewlett-Packard, Palo Alto, CA, USA) at room temperature. To reduce the contact resistance between the sintered pattern and the probe tips, a silver paste was applied at each end of the electrodes.

## 3. Results and Discussion

As the laser irradiated the precursor film, an electrically conductive pattern could be fabricated through a one-step process, which was successive nanoparticle synthesis and a sintering process. The constituting materials of the precursor were silver ions and bio-derived materials, such as Trp and gelatin. Trp played a role in the generation of nanoparticles by photoreduction through electron generation and efficient transfer of an electron to silver ions. The gelatin affected the dispersion of nanoparticles and the coating quality of the precursor for laser processing. Therefore, finding the optimal concentration of Trp and gelatin was essential. Moreover, the research to find the optimal laser processing conditions was also conducted. The study on the characteristic change of the sintering pattern, according to the laser processing conditions, was conducted. The fabrication of electrically conductive patterns was possible on the glass and PDMS. Furthermore, stable functionality of the pattern was observed against physical deformation, such as bending and twisting.

### 3.1. Synthesis of Precursor Material for Laser Sintering

First, it was necessary to consider the optimal concentration and composition of each component for nanoparticle generation with a fixed Ag concentration of 0.1 M.

#### 3.1.1. Optimal Concentration of Tryptophan in Precursor

**Figure 2:**
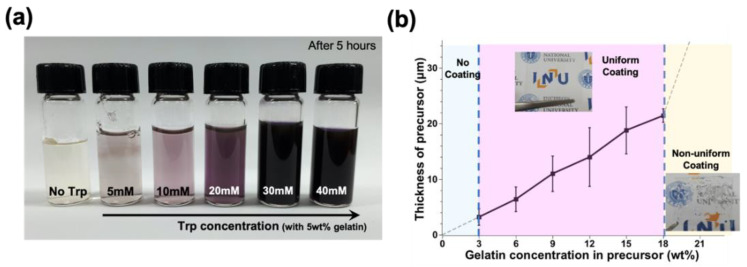
Optimal concentration of precursor composition. (**a**) Generation of nanoparticles by changing tryptophan (Trp) concentration from 0 mM to 40 mM, and (**b**) change in precursor coating thickness due to change in gelatin concentration from 3 wt% to 18 wt%.

The optimal concentration of gelatin and Trp in the precursor required for efficient patterning was studied in this study. To test the tendency of nanoparticle generation by changing a concentration of Trp in the precursor, the Trp concentration in the precursor was changed from 5 mM to 40 mM. It was observed that a small amount of silver nanoparticles was generated with laser irradiation into a precursor for 5 h (Figure 2a). The degree of nanoparticle generation could be visually confirmed by the color change of the solution. The higher the degree of the nanoparticle’s synthesis, the darker the solution. The formation rate of nanoparticles was relatively low, with the lower Trp concentration (below 10 mM). At concentrations between 10 mM and 30 mM, nanoparticles were synthesized efficiently with an increase in Trp concentration in the precursor. However, nanoparticle generation tendency was almost no different at higher Trp concentrations (30 mM) (Figure 2a). Therefore, the precursor containing 30 mM of Trp was used for efficient nanoparticle generation in this study. 

#### 3.1.2. Optimal Concentration of Gelatin in Precursor

Gelatin plays a major role in the stabilization and dispersion of the generated nanoparticles. Silver ions in precursor react with gelatin and form a stable gelatinous complex: ${\left[\mathrm{Ag}\left(\mathrm{gel}\right)\right]}^{+}\left(\mathrm{aq}\right)$

(1) $${\mathrm{Ag}}^{+}\left(\mathrm{aq}\right)+\mathrm{gel}\left(\mathrm{aq}\right)\to {\left[\mathrm{Ag}\left(\mathrm{gel}\right)\right]}^{+}\left(\mathrm{aq}\right)$$

Moreover, gelatin also played an important role in controlling the thickness and uniformity of the precursor coating. The thickness of the precursor coating mainly affected the quality and electrical conductivity of the pattern. Coating thickness was also closely related to the shape of the pattern, such as height, width, and adhesion between the pattern and the substrate during the laser processing process. 

The electrode thickness and the adhesion between the electrode and the substrate had a trade-off relationship with each other. The precursor layer absorbed optical energy at a local region near the surface. This characteristic was a fundamental limitation in performing sintering using a laser. In the case of patterning using a thick coating, a large portion of the laser energy was used for sintering, and the remaining energy was not sufficient to induce adhesion between the electrode and the substrate. This resulted in incomplete or weak adhesion of electrodes on the substrate. Therefore, control of coating thickness was important for successful patterning. The research on coating tendency according to the gelatin concentration of precursor was required for optimal laser-based fabrication. In general, as the gelatin concentration in the aqueous solution increased, the viscosity of the precursor also increased, which meant that it was possible to obtain a uniform thickness film by controlling the gelatin concentration. At the proper gelatin concentration in the precursor, some gelatin remained on the substrates due to its viscosity after the spin coating, resulting in a thin film coating. 

With a fixed concentration of Trp at 30 mM, the concentration of gelatin was changed from 0 wt% to 21 wt% in the precursor with 3 wt% intervals, and the tendency of the coating was grasped. If there was no gelatin in the precursor or the concentration of gelatin was lower than 3 wt%, the coating was impossible. All precursors from the surface were removed during spin coatings due to low viscosity. On the contrary, the viscosity became too high to coat the precursor uniformly when the concentration of gelatin was over 18 wt%. The precursor was transformed into a type of gel rapidly, and coated with a nonuniform/thickness at ambient temperature (Figure 2b). Furthermore, mixing with the silver ion aqueous solution was difficult. This also prevented the generation of uniform nanoparticles, making it difficult to fabricate the uniform/stable pattern. 

The film thickness was measured according to the change of gelatin concentration after spin coating at 2000 rpm for 15 s. The coating thickness of the film was changed from 3 μm to 21.5 μm, corresponding to the concentration of gelatin that changed from 3 wt% to 18 wt% in the precursor. As the gelatin concentration in the precursor increased, the thickness of the precursor film tended to increase (Figure 2b). However, it was impossible to measure the coating thickness with gelatin concentration exceeding 21 wt% or less than 3 wt%. If the thickness of the coating was less than 21.5 μm, the sintered pattern was well maintained after the development process. However, it showed the detachment of pattern on the substrate during postprocessing when the thickness exceeded 21.5 μm. It was affected only near the surface and not the entire depth direction of the coating during the laser processing, in the case of the thick coating. Even though, it looked as though a pattern was formed, however, the patterns were washed off during the development due to the weak attachment between pattern and substrate. The interaction due to the ratio of the proper concentration of gelatin and Trp was strongly related to efficient nanoparticle generation and the uniform precursor coating, which ultimately affected the integrity of the pattern fabrication. Therefore, the concentration of gelatin in the precursor was selected as 15 wt%, and Trp concentration was selected as 30 mM, considering the efficient nanoparticle generation and the pattern stability (e.g., adhesion) in this study. Moreover, the thickness of the coating was 18.8 μm.

### 3.2. Synthesis of Nanoparticles and Characterization

**Figure 3:**
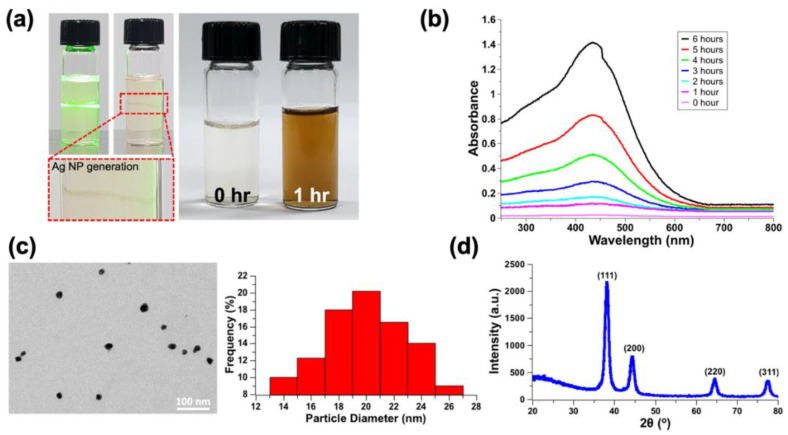
Characterization of precursor. (**a**) Generation of silver nanoparticles by laser irradiation, (**b**) absorption spectra of precursor for silver nanoparticle generation, (**c**) TEM image and particle size distribution, and (**d**) result of high-resolution X-ray diffraction (HR-XRD).

It was observed that nanoparticles were generated along the laser beam path. In addition, it also was confirmed, through the color change in the solution, that there was nanoparticle generation after laser treatment for 1 h (Figure 3a).

The formation of silver nanoparticles was monitored using UV–Vis absorption spectra in wavelengths ranging from 250 to 800 nm. Figure 3b shows the absorption spectrum of UV–visible light in the laser-irradiated sample with time and a bare sample as reference. Silver nanoparticles have free electrons that yield a surface plasmon resonance (SPR) absorption band due to the mutual vibration of the silver nanoparticle electrons in resonance with a light wave [27,28]. The shape of the peak represents the surface plasmon resonance of silver nanoparticles. The increase in intensity of the plasmon band could be predicted through the decrease in the bandwidth [29]. In addition, it was observed that the surface plasmon peak occurring at 440 nm slowly shifted to a low wavelength (433 nm) at high concentrations. This shift may have been due to the blue shift and depended on particle size and shape. Therefore, efficient silver nanoparticles were confirmed by laser irradiation to the precursor [30,31].

The generation and size of nanoparticles were confirmed through transmission electron microscopy (TEM). The diameter of the nanoparticles, which were synthesized using the precursor material with an optimal concentration of gelatin and Trp, was measured through TEM. After the image processing of the TEM image, it was confirmed that the diameter of the nanoparticles was in the range of 14 nm to 26 nm, and it could be concluded that a uniform nanoparticle was generated (Figure 3c).

After irradiating the precursor material, the X-ray diffraction (XRD) data confirmed that the silver nanospheres were crystalline, with a face-centered cubic (FCC) structure. The precursor film was processed using 90 mW, 5 mm/s of laser processing condition after spin coating on glass substrate at 2000 rpm for 15 s. To remove a residual solvent after the washing process, the 10 mm × 10 mm sintered film was dried in the ambient air for 10 min before XRD measurement. The XRD data showed sharp peaks at 2*θ* = 38.2°, 44.5°, 64.56°, and 77.54°, which corresponded to the (111), (200), (220), and (311) planes, which were FCC crystal structures. (Figure 3d).

### 3.3. Optimal Condition for Laser Processing

**Figure 4:**
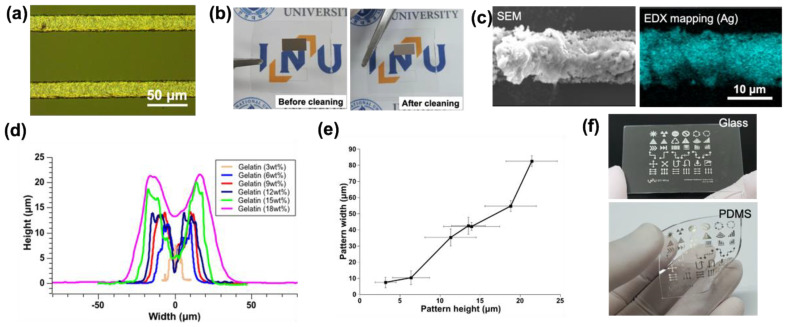
Patterning process using laser-direct writing. (**a**) Optical image of a sintered pattern on the glass, (**b**) image before/after postprocessing after laser processing, (**c**) SEM image and element mapping image using SEM-EDX, (**d**) cross-sectional profile of the pattern by surface profiler, (**e**) relationship between height and width of the pattern, and (**f**) diverse shape pattern on glass and PDMS.

To find optimal laser fabrication conditions, the thickness of the coating, the laser power, and the scan speed should be considered, as the maximum temperature rise at the surface is affected by those factors (Supplementary Materials). As previously described, the precursor coating thickness is closely related to the gelatin concentration. If the concentration of gelatin was low, the thickness of the precursor was thinly coated. In this case, laser irradiation directly affected the precursor film even when processing with a low laser density. The central part of the pattern tended to be deeper than the peripheral part (Figure 4d). When the laser density increased, ablation occurred in the center, which could cause serious damage to the film and pattern. If the concentration of gelatin was sufficient but the concentration of Trp was low, then metal nanoparticles were not synthesized sufficiently and it was not possible to fabricate a well-connected metal pattern. The relatively high concentration of the organic compound in the precursor was thermally decomposed and generated the gas during the laser process. Moreover, it was observed that the tendency of the porosity increased as the concentration of organic compounds increased (Figure S1). This induced a drop in electrical conductivity due to the imperfect connectivity of the pattern. Moreover, this was not suitable for use as an electrode. Therefore, 15 wt% of optimal concentration of gelatin in the precursor was used, and its thickness was 18.8 μm.

#### 3.3.1. Optimal Condition for Sintering on Glass

The parametric study was conducted to find the optimal sintering conditions by changing the energy density and scanning speed with fixed gelatin concentration to minimize the deformation (e.g., ablation, melting) of the substrate.

For the parametric study on the glass substrate, the scan speed was changed from 2 mm/s to 11 mm/s at intervals of 1 mm/s, and the laser power was changed from 10 mW to 170 mW at intervals of 10 mW (Supplementary Materials). The pattern quality was checked by measuring the appearance and electrical conductivity of 10 samples for each condition. The lowest sheet resistance (22.4 Ω sq^−1^) was measured in a pattern processed using a scan speed of 5 mm/s and an energy density of 2.4 J/cm^2^ (Figure S2b). When grid shapes with different spacing were fabricated using the optimal conditions, the sheet resistance tended to increase as the pitch of the mesh grid was increased (Figure S2c).

The fact that the precursor material was based on an organic material represented the unique properties. It is worth noting that a low laser density was required for patterning. Organic materials could be carbonized, or damage to the pattern and substrate could occur under high laser power density. However, it was advantageous to obtain a more sophisticated shape than that using conventional nanoparticle-based inks. The selective laser sintering was based on a photothermal process. The pattern could be fabricated based on the temperature distribution produced by the focused laser beam and the heat transfer mechanism. The size of the pattern was related to thermal diffusion by the nanoparticle itself with high thermal conductivity. When sintering was performed using organic material-based ink, relatively small thermal absorption and thermal diffusion were induced, due to the lower thermal conductivity. This resulted in relatively uniform thermal diffusion, making it possible to fabricate a smaller pattern. Ideal patterning was possible when patterning was performed using 90 mW, and the laser power density was approximately 40.8 W/cm^2,^ considering the laser beam diameter was about 750 μm. It was confirmed that this laser power density had similar sintering conditions to previous studies [32]. 

#### 3.3.2. Pattern Fabrication and Characterization (Pattern on Glass)

The pattern fabricated under these experimental conditions had a smaller shape (50 μm) than the size of the laser beam (750 μm) (Figure 4a,d). However, there were still limitations to be solved. The efficiency of nanoparticle formation and sintering was significantly reduced using lower laser output than a threshold. Moreover, it was a limitation in that the slower fabrication speed, than that of using conventional synthesized silver nanoparticles, resulted in a longer processing time. The whole process was based on a one-step process in which the nanoparticle generation and sintering process occurred simultaneously. Therefore, a sufficient conversion process from silver ions to nanoparticles was required, and a large number of generated particles should be sintered for successful patterning. 

After the laser-based machining was completed, the removal of the non-sintered region was important for electrically conductive pattern fabrication, as the electrical conductivity was lower when the non-sintered region was not reliably removed. Since all the constituent materials of the precursor were composed of water-soluble material, it was easy to remove the non-sintered region using DI water without harsh chemical treatment. Figure 4b shows a pattern sample before/after the cleaning process. It suggests that the precursor used in this study had the strength to enable eco-friendly postprocessing. 

The elemental mapping images for patterns could be obtained through SEM and SEM-EDX (Figure 4c). From the elemental mapping results of SEM-EDX, it was confirmed that only the pattern region showed the properties of silver. 

The morphology of the pattern was measured using a surface profiler. As the laser was irradiated during processing, the material and the generated nanoparticles moved from the center to the periphery, due to the thermocapillary phenomenon, resulting in a binodal cross-sectional shape similar to a high volcanic shape (Figure 4d). The width of the pattern was closely related to the thickness of the precursor coating. The higher laser power and longer processing time were required to enable pattern formation and adhesion to the substrate with a thicker coating. Thermal energy in the thin coating film after laser irradiation could be transferred not only in the vertical direction of the surface but also in the lateral direction simultaneously. Therefore, the width of the pattern increased as the height of the pattern increased (Figure 4e).

#### 3.3.3. Optimal Condition for Sintering on PDMS

In this study, the patterning on PDMS was also conducted, in that it satisfied high electrical conductivity and transmittance simultaneously. The sintering process on elastomer substrates, such as PDMS, is a challenge due to the poor wettability of PDMS and with discrepancies between the PDMS and the metal nanoparticles concerning their mechanical properties [33]. Existing studies have sintered silver nanoparticles on the PDMS using a capillary-assisted, laser-direct writing (CALDW) method, but multiple overlapping scanning methods were used for high electrical conductivity [34]. Due to the properties of the precursor, which was an organic material-based material, lower laser power was required for the generation of nanoparticles and laser processing at low temperatures. Due to these characteristics, pattern fabrication was possible on a PDMS substrate. 

The parametric study was performed to obtain optimal laser power and scan speed in the same manner as that on the glass. (Supplementary Materials, Figure S3a). From the results of the parametric study, the required optimal energy and scan speed were 35 mW and 3 mm/s, respectively (Figure S3b). The electrode pattern formed using these optimal processing conditions was stably maintained on the substrate and had excellent electrical conductivity. To measure the electrical conductivity and apply the voltage to the pattern, the silver paste was applied to both ends of the pattern on the PDMS and then connected by a copper wire. Moreover, PDMS was re-coated again using a spin coater to prevent the secondary deformation and oxidation of the pattern. The laser power required for this process was relatively low compared to that for processing on glass. In addition, the processing speed was also relatively slow compared to that for processing on glass. 

Theoretically, the required laser power, depending on the substrate materials, can be described in the following equation, which is based on the theory of transient diffusive heat transfer in a semi-infinite solid with constant surface heat flux as $q_{s}^{\prime \prime }=q_{0}^{\prime \prime }$ [35].

$$T\left(x,t\right)=T_{i}+\frac{2q_{0}^{\prime \prime }{\left(\alpha t/\pi \right)}^{1/2}}{k}\exp\left(\frac{-x^{2}}{4\alpha t}\right)-\frac{q_{0}^{\prime \prime }x}{k}erfc\left(\frac{x}{2\sqrt{\alpha t}}\right)$$

where $T_{i}$, x, t, α, and k correspond to the initial temperature, the depth from the surface, time, thermal diffusivity, and thermal conductivity of the substrate, respectively. Assuming that the thickness of the precursor coating was very small (t≈0) compared to that of the substrate, temperature increase ($T-T_{i}$) at the surface (x=0) is proportional to the value of $\alpha^{1/2}/k$. The thermal properties of glass and PDMS are shown in Table 1. Considering the value of $\alpha^{1/2}/k$ of PDMS is about three times higher than that of glass (Table 1), it can be concluded that the required laser output for patterning on PDMS is lower than that of glass. The optimal power density is 40.8 W/cm^2^ for glass, and 15.87 W/cm^2^ for PDMS.

The processing speed is also relatively slow compared to that for processing on glass (Supplementary Materials, Table S1). When the scan speed is set high, the bonding force between the pattern and the substrate is weak, which does not maintain the pattern on a substrate during the development process. When the scan speed is set high, the bonding force between the pattern and the substrate is weak, which does not maintain the pattern on a substrate during the development process. 

#### 3.3.4. Pattern Fabrication and Characterization (Pattern on PDMS)

The change in transmittance of the grid-type pattern also was measured according to the wavelength from 400 nm to 800 nm. In the pattern of the same pitch, the transmittance was almost uniform, but the transmittance tended to increase as the pitch increased (Figure S4b). At a specific wavelength (550 nm), not only sheet resistance but also transmittance tended to increase at the same time as the pitch increased (Figure S4c). 

Furthermore, it was possible to fabricate arbitrary shapes patterns using a galvano scanner as well as general line patterns (Figure 4f). The diverse shape of pattern fabrication showed the possibility that it could be utilized in various applications.

### 3.4. Mechanical Flexibility/Stability and Electrical Reliability of the Pattern

#### 3.4.1. Mechanical and Electrical Performance Tests of Straight Meshed-Type Pattern

First, the mechanical flexibility/stability and electrical reliability of the pattern against the deformation were tested by cyclic bending and the twist test.

**Figure 5:**
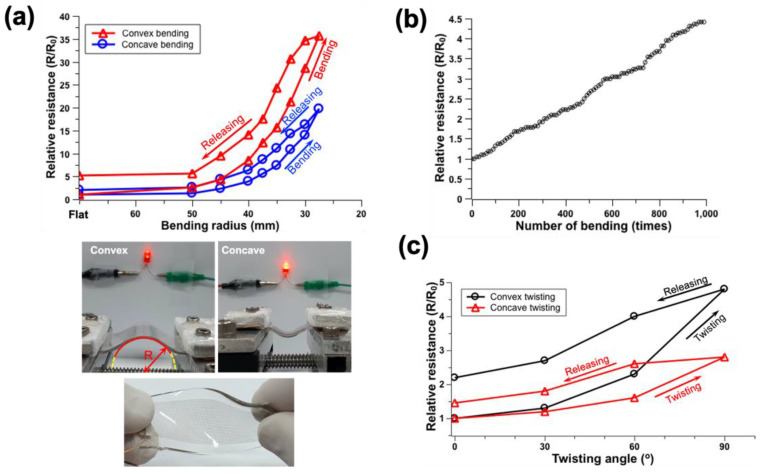
Mechanical and electrical performance tests of straight meshed-type pattern on PDMS. (**a**) Relative resistance change by different bending radius change, (**b**) relative resistance change in repeated bending with a bending radius of 55 mm, and (**c**) relative resistance change by different twisting angle.

The straight-type meshed patterns with 700 μm spacing on the PDMS substrate were fabricated. For the bending test, the sample was mounted and fixed to the fixed part, and the moving part at the other side of the mechanical linear rail. The position was adjusted using the Arduino controller (ATMega328) to control the bending radius and repetition time. The curvature radius of the pattern was set from 50 mm to 27.5 mm and measured the changes of relative resistance (R/R_o_) where R_o_ represents the resistance in the original state, and R is the resistance after deformation (Figure 5a). As the radius of curvature in the bending process was smaller, the relative resistance increased. As the radius of curvature was set to 27.5 mm in the convex mode, the relative resistance increased sharply by approximately 35 times.

In addition, the stability of the pattern for the bending direction was also tested. The pattern was subjected to opposite load depending on this bending direction (Figure 5a). When the substrate was bent in the convex direction, the pattern was subjected to a tensile load. In contrast, a compressive load was applied to the pattern when the substrate was bent in the concave direction. The resistance tended to increase in both the concave and convex bending modes. The increase in electrical resistance in the convex bending was significantly bigger than that of the concave bending. A circuit including a light-emitting diode (LED) was made to visualize the resistance change during the bending test. The significant blinking of the connected LED to the circuit was observed during the convex bending mode (Figure 5a). In the concave mode bending, the relative resistance increased by approximately 20 times at a bending radius of 27.5 mm. The electrical resistance in the convex mode was higher than that in the concave mode, even when returning to the original state. The relative resistance increased by approximately 105% in the concave mode bending when returning to the flat state, whereas it was maintained at an increase of approximately 412% in the convex mode bending. This was expected because the tendency of microcrack generation in the convex mode bending was higher than that in concave mode bending (Figure S5).

To test the stability of the pattern against repeated bending, one end of the pattern was fixed and the other end was moved at a frequency of 1 Hz (Figure 5b). The curvature radius of the bent substrate was set to 55 mm. The resistance was measured in the flat state after 10 bending cycles. The relative resistance (R/R_o_) increased up to approximately 4.6 times (from 25 Ω to 115 Ω) of the original value constantly after the repeated bending 1000 times. This phenomenon was caused by the formation of microcracks. If bending was repeated more than 2000 times, the resistance increased to infinity due to the short circuit of the electrode. 

This phenomenon also can be explained by the tunneling theory. Electrical conductivity is related to the distance between the conductor (e.g., pattern or particles). The electrons can pass to an adjacent conductor part when there is a small gap between the microscale cracks. Hence, a conductive channel can be formed even without direct contact. When the bending state returns to its original state, part of the resistance is restored. However, as the gap between the electrodes is increased, the tunneling effect is also sharply decreased, leading to the resistance increase. When repeated bending is continued, the electrode is shorted and the electrical conductivity becomes zero.

In addition, mechanical/electrical reliability by twist was also affirmed. The change of relative resistance was measured by changing the twist angle from 30° to 90° at 30° intervals (Figure 5c). As the twist angle increased, the change in resistance was also increased. In the concave mode twist, the relative resistance did not increase significantly as the angle increased to 60°. In contrast, the relative resistance increased up to 2 times in the convex mode twist. The electrical resistance showed a tendency to increase regardless of being convex or concave, but a larger change in resistance was measured in convex, as in the bending experiment. It was confirmed that the relative resistance increased by approximately 150% and 225% in the convex and concave twisting mode, respectively, when it returned to the flat state again. Similar to the bending test, this was expected to be due to the generation of discontinuities as microcracks during the twisting. On the LED setup, a significant flickering of the LED was not observed until a twist of 90°, however, the light intensity of the LED sharply decreased in the twist at more than 90°.

#### 3.4.2. Mechanical and Electrical Performance Tests of Fractal Pattern

As the straight-line electrode pattern on the PDMS was relatively vulnerable to bending and twisting, the fractal structure was adopted as a new approach to overcoming this limitation (Figure S6a). A fractal structure can be described by self-similarity, when the geometry of the subdivisions resemble that of the whole structure [38]. This fractal structure has a variety of shapes from lines (Peano, Hilbert, Koch) to loops (Vicsek, Moore) and branch-like meshes (Greek cross). Fractal designs can be engineered to accommodate enhanced elastic strain, and resist diverse deformation to support biaxial, radial directions. This structure is suitable for use in various stretchable devices such as the epidermal electronic platform [39]. 

**Figure 6:**
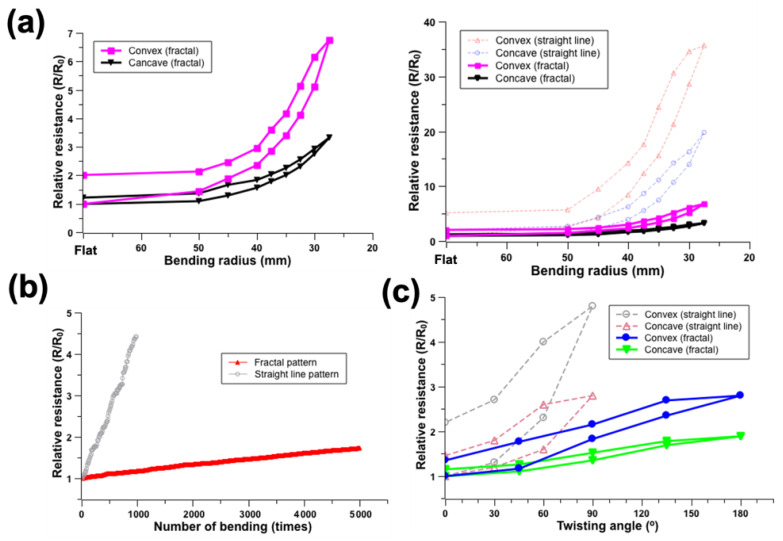
Mechanical and electrical performance tests of fractal patterns on PDMS. (**a**) Relative resistance change by different bending radius change (**left**) and comparison of relative resistance change between straight and fractal-shape patterns (**right**), (**b**) relative resistance change in repeated bending with a bending radius of 55 mm and comparison of relative resistance change between straight and fractal-shape pattern, and (**c**) relative resistance change and comparison of relative resistance change between straight and fractal-shape pattern by different twisting angles.

Bending tests and twist experiments were performed in the same manner after fabricating a mesh-type fractal pattern structure (Figure S6b). In the bending experiment of the fractal structure pattern, as the radius of curvature decreased, the relative resistance showed a trend to increase. However, the resistance change was smaller than that in the straight-structured, pattern-bending test (Figure 6a). In addition, the relative resistance change, according to the bending direction, showed a similar result in the straight-line bending test. Relative resistance change in the convex bending mode was bigger than that in the concave bending mode. In addition, when it was returned to the original, flat state, the increase in the relative resistance in the fractal structure was smaller than that in the straight line.

The relative resistance also showed a tendency to increase with an increase in the number of repetitions of bending (Figure 6b). Although the number of repetitions of bending in the fractal structure pattern was greater than that in the straight structure pattern, the resistance increase was smaller. Similar to the bending test, the change amount of resistance in the fractal structure was smaller than that in the straight pattern with a change in twisting angle (Figure 6c). In addition, it was observed that the structure could resist up to 180 degrees, which was a larger withstanding twisting angle of the straight-type pattern. Even when the pattern was returned to an original state, the increase in the relative resistance in the fractal structure was significantly smaller than the increase in the relative resistance in the straight-type pattern. Therefore, it can be concluded that fractal structures can resist mechanical deformation (e.g., bending, twisting) more efficiently than straight patterns.

While applying physical deformation (e.g., bending and twisting), the electrical property change of the pattern was measured. The electrical conductivity of the straight-type meshed pattern on the PDMS was significantly changed, even though there was only a small amount of bending and twisting. This limitation could be overcome through the introduction of tensile-resistant fractal structure patterning. Compared to the straight-lined pattern, the fractal structure showed efficient resistance to external physical deformations, such as bending and twisting.

### 3.5. Adhesion Test for Mechanical Stability between the Pattern and Substrate

The adhesion between the pattern and substrate was tested through tape-pull and ultrasonication tests (Figure S7). The large-area pad for the adhesion test was fabricated on the glass and PDMS using optimal conditions (90 mW at 5 mm/s and 35 mW at 3 mm/s for glass and PDMS, respectively). 

First, the tape-pull test was conducted by repeating the action of attaching and peeling off a commercial adhesive tape (Scotch^®^ MagicTM tape, 3M) on a large-area pad on glass and PDMS several times (Figure S7a). As a result, the large-area pad pattern was not separated from the substrate, which could confirm that no separated pattern was on the detached tape.

Next, another adhesion test was performed using an ultrasonic bath (Daihan scientific, Wonju, Korea). The stability of the adhesiveness of the pattern was tested by applying ultrasonic waves, this was performed by immersing the patterns on the glass and PDMS substrate into the ultrasonic bath for 60 s (Figure S7b). As a result, the pattern was stably maintained without damage or detachment from the substrate after ultrasonic treatment. Moreover, no damage or detachment of the pattern was observed even though ultrasonication was repeated (over 10 times).

The pattern fabricated through laser-based sintering was stably maintained without damage or detachment from the surface, even under the tape-pull and ultrasonic conditions. This suggested the possibility for large-area processing on various substrates with suitability for a diverse range of applications.

### 3.6. Limitations and Future Work

There are still limitations to fabricating the electrically conductive electrodes through laser-based sintering of the nanoparticles, which are synthesized using the organic/solution-based inks. Considering the material aspect, since organic substances dissolved in the precursor ink, it generated the porous structure due to the heat generated during the process. This structure resulted in a decrease in the electrical conductivity of the pattern. In addition, since the synthesis and sintering of nanoparticles must be performed simultaneously in the processing process, the fabrication speed was relatively low compared to the conventional patterning process of sintering the already-synthesized nanoparticles directly. Despite these limitations, it was meaningful considering that all compositions of ink are from nature, and un-sintered parts can be removed with DI water as a new, eco-friendly electrode processing method. 

As previously described, the relatively small thermal absorption and thermal diffusion of organic material-based ink resulted in the smaller pattern fabrication. 

Considering the processing method using a different kind of laser, the patterning using a femtosecond laser enabled high-resolution metal patterning through the reaction between the femtosecond laser and the nanoparticles [40]. This overcame the diffraction limit of the laser due to the nonlinear optical effect, enabling a pattern with a size smaller than the wavelength of the light source. Although this had a limitation in that the processing speed was slow, meaning the processing time was long. However, through the grafting of the organic/solution-based inks used in this study with femtosecond laser-based sintering, extensions to new application fields can be expected.

## 4. Conclusions

As interest in the environment increases, the demand for environmentally friendly manufacturing has increased. However, there are limitations in that processing using toxic substances exists in fabrication process. Therefore, studies have been conducted to synthesize nanoparticles in an eco-friendly way and to apply them to various applications. Hints from nature can also provide clues to solving problems. In this study, the efficient electron transfer mechanism of Trp was adopted. It was possible to synthesize silver nanoparticles through photoreduction of metal ions by adopting the efficient electron transport mechanism in the presence of Trp under laser irradiation. However, the ink and the mixture of the Trp and silver ion, could not be coated on the surface for laser processing. Therefore, it was only possible to achieve those goals by mixing the appropriate ratio concentration of gelatin and Trp. It did not only stabilize the generated nanoparticles but also controlled the particle size and coating thickness. By combining this with the laser direct writing technique, digital-maskless, high-resolution, and low-temperature metal patterning was possible on various substrates. In addition, this material composition was also closely related to postprocessing (e.g., washing). Since all the materials contained in the precursor were from nature, the un-patterned region could be removed efficiently with only DI water, rather than a toxic solvent. Therefore, the entire process, from ink preparation to postprocessing, was an eco-friendly patterning process. Moreover, the excellent mechanical stability against external mechanical loads was verified by bending, twisting, and adhesion tests. A mesh-type conductive pattern with high transmittance and low sheet resistance could be fabricated based on superior electrical properties. In addition, it was also possible to fabricate a bendable, electrically conductive platform by patterning the fractal structure. In this study, efficient and eco-friendly electrically conductive pattern fabrication was possible through the organic/solution-based inks, which were inspired by the unique mechanisms of nature, and the eco-friendly, laser-based patterning techniques.

## Figures and Tables

**Table 1:** Thermal properties of glass and PDMS.

	Thermal Diffusivity $\alpha \left[m^{2}/s\right]$	Thermal Conductivity k[W/m K]	$\alpha^{1/2}/k$
Glass [35] PDMS [36,37]	3.4 × 10^−7^ 0.7 × 10^−7^	1.06 0.15	5.5 × 10^−4^ 1.75 × 10^−3^

## Data Availability

The data presented in this study are available in this article.

## Supplementary Materials

The following Supplementary Materials can be downloaded at: https://www.mdpi.com/article/10.3390/nano12030545/s1. Figure S1: Change in porosity of pattern by the change in gelatin concentration from 3 wt% to 18 wt% and different concentrations of tryptophan; Figure S2: Parametric study for optimal laser processing on the glass. (a) Patterning result according to laser power and scanning speed, (b) sheet resistance change by changing the laser power at fixed scanning speed (5 mm/s), and (c) sheet resistance change with different pitch of grid pattern with 85 mW laser power and 5 mm/s scanning speed; Figure S3: Parametric study for optimal laser processing on the PDMS. (a) Patterning result according to laser power and scanning speed, (b) sheet resistance change by changing the laser power at fixed scanning speed (3 mm/s), and (c) sheet resistance change with different pitch of grid pattern with 35 mW laser power and 3 mm/s scanning speed; Table S1: Optimal fabrication parameter for high electrical conductivity; Figure S4: Transmittance spectra of the meshed pattern on PDMS. (a) Optical image of the meshed-type pattern, (b) transmittance result with different pitches of pattern, and (c) relation between transmittance and sheet resistance at different pitches; Figure S5: Microscale crack generation after the repeated bending; Figure S6: Fractal structure fabrication for durability against physical/mechanical deformation. (a) Schematic of fractal structure and geometry of subdivision, and (b) image of fabricated fractal structure; Figure S7: Adhesion test for mechanical stability between the pattern and substrate. Image of (a) tape-pull test and (b) ultrasonication test result of the pattern on glass (left) and PDMS (right).
