# Supplementary material for: Green Synthesis of Nanoparticles Using Bio-Inspired Systems and Electrically Conductive Pattern Fabrication through Laser-Direct Writing
Source: Nanomaterials (Basel). 2022 Feb 5;12(3):545. doi: 10.3390/nano12030545 (PMC8840062; doi:10.3390/nano12030545)
Supplement: Supplementary file 1 [file nanomaterials-12-00545-s001.zip › nanomaterials-1566808-supplementary.pdf]

nanomaterials

MDPI

Supplementary Material

Green Synthesis of Nanoparticles Using Bio-Inspired Systems and Electrically Conductive Pattern Fabrication through Laser-Direct Writing

Sangmo Koo

Department of Mechanical Engineering, Incheon National University, Incheon 22012, Korea; skoo@inu.ac.kr

1. Change in Porosity of Pattern by the Change in Gelatin Concentration

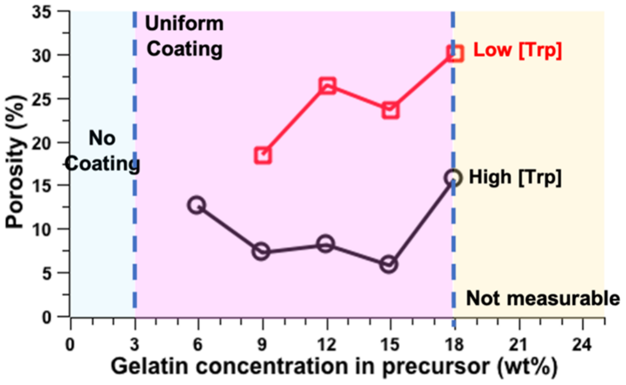

Figure S1. Change in porosity of pattern by the change in gelatin concentration from 3 wt% to 18 wt% and different concentrations of tryptophan.

2. Fundamental of the Laser-Assisted Sintering Process

By irradiating the laser light on the precursor composed of the appropriate ratio of gelatin and tryptophan, a metal conductive electrode patterning was possible on various substrates (e.g., glass and PDMS) through sequent nanoparticle generation and sintering simultaneously.

The fundamentals of laser-assisted sintering of nanoparticles are based on the coupling of photon excitation and surface plasmonic resonance of the nanoparticles. The highly localized thermal energy from the laser melts the nanoparticles and connects between nanoparticles. The affected area by thermal energy is limited only to the highly localized heat-affected zone, and it means that patterning on the flexible substrate was possible as a low-temperature process. Theoretically, the maximum temperature rise at the surface can be described for relatively slower scan speed with laser irradiation and surface absorption as follows.

$$\Delta._{max}(Z^* = 0) \simeq \frac{\theta_c}{2\sqrt{\pi} h_s^*} \ln\left(\frac{16}{\xi {v_s^*}^2}\right) \quad (1)$$

where θ_c, h_s^* and v_s^* correspond to the (normalized) laser power, the thickness of the film and the scanning speed, respectively. ξ Is a constant defined as $\exp(C+1)$ where C is 0.577 [1].

From this equation, the maximum temperature rise at the surface is affected by the thickness of the coating, the laser power, and the scan speed. Therefore, a parametric study was performed to find the optimal sintering conditions based on the change of the thickness of the coating, laser power, and scanning speed.

3. Parametric Study on Glass and PDMS Substrate

3.1. Glass Substrate

For the parametric study on the glass substrate, the scan speed was changed from 2 mm/s to 11 mm/s at intervals of 1 mm/s, and the laser power was changed from 10 mW to 170 mW at intervals of 10 mW (Figure S2a). When the laser power was low, but the scanning speed was high with the same energy density, the adhesion between the incomplete pattern and the substrate is weak. The generation and sintering of nanoparticles by laser is low when the laser power and the scan speed are simultaneously low in the case of the same energy density, resulting in incomplete pattern fabrication. The number of patterns that are lifted off during the cleaning process.

The pattern quality was checked by measuring the appearance and electrical conductivity of 10 samples for each condition. For the calculation of the power density (Power/Area) of a laser beam

$$. \ ower\ density\ (W/cm^2) = \frac{255}{d^2} \times Power \quad (2)$$

where d is the diameter of the laser beam in millimeters. When the energy density was lower than 18.1 W/cm^2, the pattern was not fabricated well. The fabricated patterns were well-connected from 18.1 W/cm^2 to 81.6 W/cm^2 of the energy density. However, the electrical conductivity tends to decrease rapidly when the energy density exceeds 81.6 W/cm^2.

The damage of the sintered pattern is induced when energy exceeding required for sintering is applied, leading to a sharp drop in electrical conductivity (Figure S2b).

To confirm the applicability to the electrode, the sheet resistance (R_s) according to the pitch of the patterns was measured (Figure S2b). The resistance was measured on two sides of each of the 50mm x 50mm mesh patterns using two-terminal methods. The sheet resistance can be calculated by

$$R_s = \frac{RW}{L} \quad (3)$$

where R, W, and L are resistance, width, and length of the mesh-type panel, respectively. As the pattern spacing increases, the sheet resistance tends to increase (Figure S2c). The resistivity of the pattern was calculated using ϱ = R(A/l), where R, A, and l are the resistance, cross-section area, and length of a single electrode line, respectively. The lowest resistivity was approximately 340 nΩ m, which is approximately 21.3 times higher than bulk Ag (15.9 nΩ m at 20 °C) [2]. These results are expected to originate from the nanoscale pore formation, partial oxidation, incomplete reduction, and insulator residues after development due to organic material-based characteristics in the pattern fabrication. The highest relative conductivity was measured in a pattern processed using a scan speed of 5 mm/s and energy density of 40.8 W/cm^2. (Figure S2b).

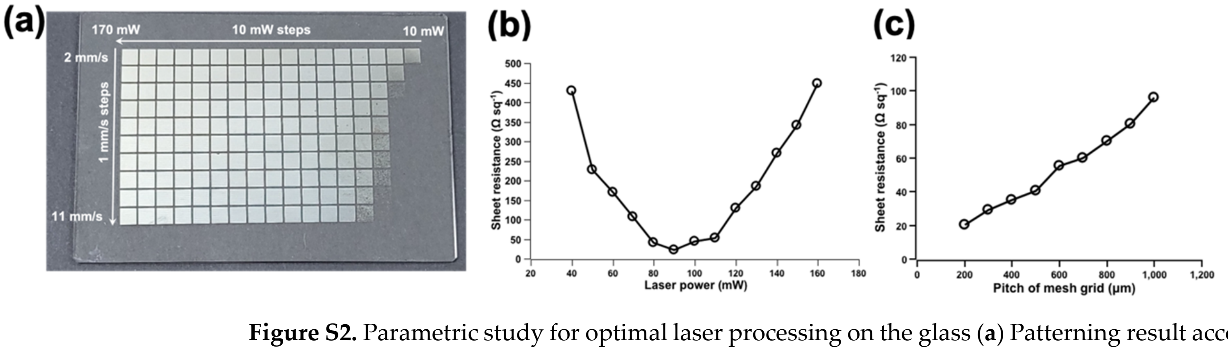

Figure S2. Parametric study for optimal laser processing on the glass (**a**) Patterning result according to laser power and scanning speed, (**b**) Sheet resistance change by changing the laser power at fixed scanning speed (5mm/s), and (**c**) Sheet resistance change with different pitch of grid pattern with 85mW laser power and 5mm/s scanning speed.

3.2. PDMS Substrate

For the parametric study on the PDMS substrate, the laser power was changed from 15 mW to 65 mW at intervals of 10 mW, and the scan speed was changed from 2 mm/s to 5 mm/s at intervals of 1 mm/s. However, the pattern was severely damaged where the laser power was exceeded 45 mW, and the pattern was in-completed where the laser power was lower than 25 mW. Therefore, the range and interval of the laser power and the scan speed are subdivided, the laser power was changed from 25 mW to 45 mW at intervals of 4 mW, and the scan speed was changed from 2 mm/s to 4.5 mm/s at intervals of 0.5 mm/s (Figure S3a). The appearance and electrical conductivity of 10 samples were measured to check the pattern quality. When the energy density was lower than 12.92 W/cm^2, the pattern was not fabricated. The fabricated patterns were well-connected from 12.92 W/cm^2 to 20.4 W/cm^2 of the energy density. However, the electrical conductivity tends to decrease rapidly when the energy density exceeds 20.4 W/cm^2 due to the damage (Figure S3b).

The sheet resistance (R_s) according to the pitch of the patterns on PDMS was measured. The resistance was measured on two sides of each of the 50mm × 50mm mesh patterns using two-terminal methods. The sheet resistance of the grid pattern which is fabricated using the optimal conditions with different spacing was measured. It was observed that the sheet resistance tends to increase as the pitch of the mesh grid was increased (Figure S3c). The lowest resistivity was approximately 212.8 nΩ m, which is about 13.4 times higher than bulk silver (15.9 nΩ m at 20 °C) [2]. These results are also expected to originate from the porosity of pattern, partial oxidation, incomplete reduction, and insulator residues. The highest relative conductivity was measured in a pattern processed using a scan speed of 3 mm/s and energy density of 15.87 W/cm^2. (Figure S3b).

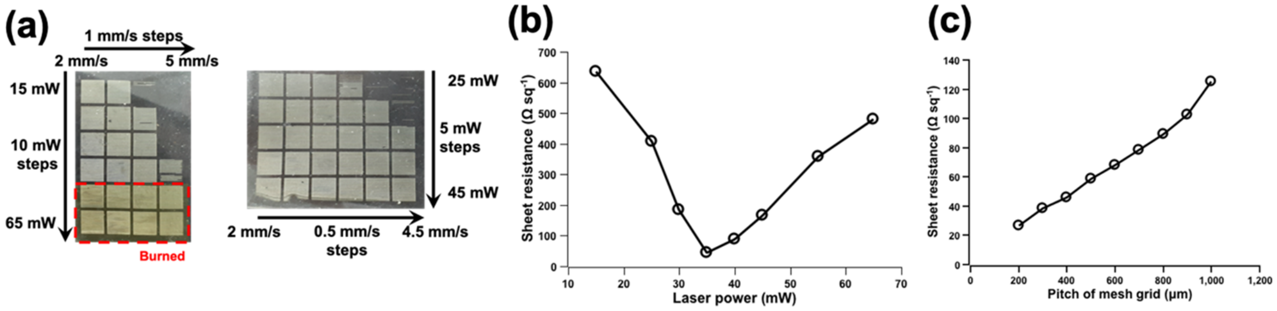

Figure S3. Parametric study for optimal laser processing on the PDMS (**a**) Patterning result according to laser power and scanning speed, (**b**) Sheet resistance change by changing the laser power at fixed scanning speed (3 mm/s), and (**c**) Sheet resistance change with different pitch of grid pattern with 35 mW laser power and 3 mm/s scanning speed.

Table S1. Optimal fabrication parameter for high electrical conductivity.

	Laser power (mW)	Scan speed (mm/s)
Glass	90	5
PDMS	35	3

4. Transmittance Spectra of the Meshed Pattern on PDMS

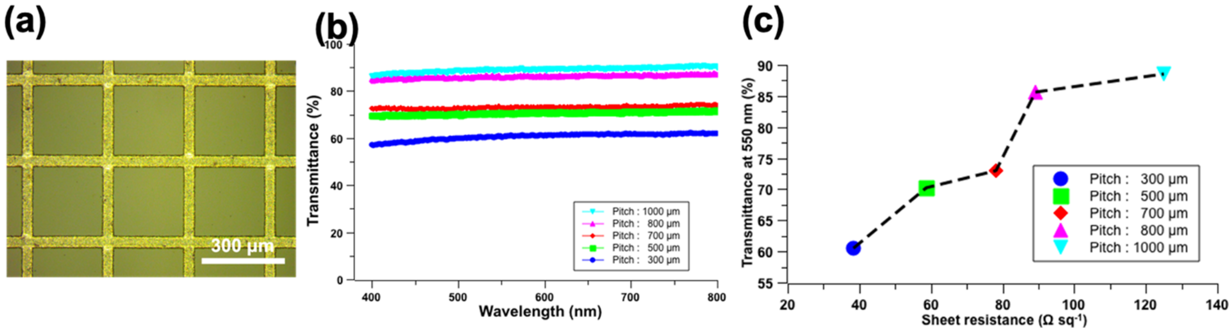

Figure S4. Transmittance spectra of the meshed pattern on PDMS (**a**) Optical image of the meshed-type pattern, (**b**) Transmittance result with different pitches of pattern, and (**c**) Relation between transmittance and sheet resistance at different pitches.

5. Microscale Crack Generation

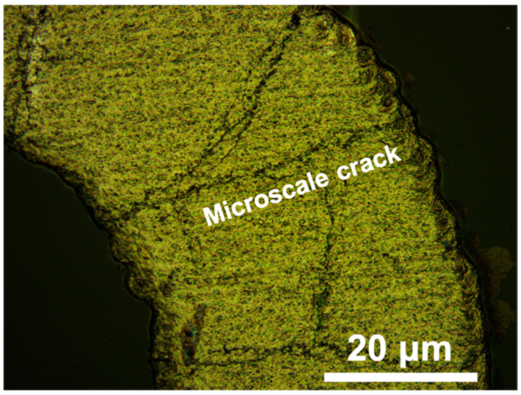

Figure S5. Microscale crack generation after the repeated bending.

6. Fractal Structure Fabrication

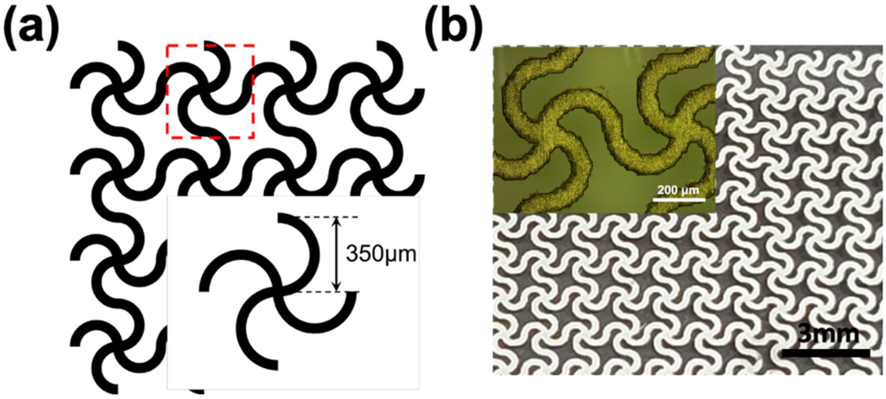

Figure S6. Fractal structure fabrication for durability against physical/mechanical deformation (**a**) Schematic of fractal structure and geometry of subdivision, and (**b**) Image of fabricated fractal structure.

7. Adhesion Test For Mechanical Stability Between the Pattern and Substrate

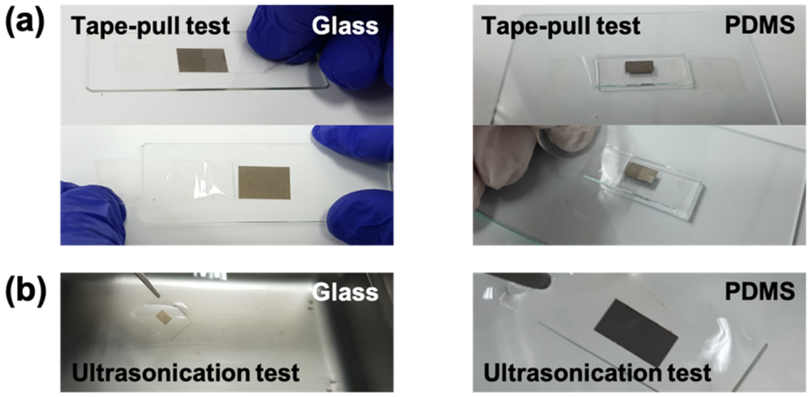

Figure S7. Adhesion test for mechanical stability between the pattern and substrate. Image of (**a**) tape-pull test and (**b**) ultrasonication test result of the pattern on glass (left) and PDMS (right).

References

1. Baüerle, D. In *Laser Processing and Chemistry*; Springer: Berlin, Heidelberg, Germany, 2011.
2. Matula, R.A. Electrical resistivity of copper, gold, palladium, and silver. *Journal of Physical and Chemical Reference Data* **2009**, *8*, 1147, doi:10.1063/1.555614.
